# Maladie de Haglund: à propos de trois cas

**DOI:** 10.11604/pamj.2015.22.37.7866

**Published:** 2015-09-17

**Authors:** Amégninou Mawuko Yao Adigo, Néille Gbèssi Gnakadja, Yaovi Yanick Dellanh, Kokou Adambounou, Oni Djagnikpo, Lama Kegdigoma Agoda-Kousséma, Abikou Léon Adoko, Komlanvi Victor Adjénou

**Affiliations:** 1 Service de Radiologie du CHU Campus, Lomé, Togo; 2 Service de Traumatologie-Orthopédie du CHU Sylvanus Olympio, Lomé, Togo; 3 Service de Radiologie du CHU Sylvanus Olympio, Lomé, Togo

**Keywords:** Maladie de Haglund, écho-Doppler, radiographie, IRM, Afrique, Haglund deformity, Doppler, radiograph, MRI, Africa

## Abstract

La maladie de Haglund est une pathologie relativement sous évaluée. Elle est liée à un conflit calcanéo-achilléen. Nous rapportons les cas de patients âgés de 40, 42 et 37 ans, révélés par des œdèmes douloureux de la cheville. Le diagnostic a été confirmé à la radiographie standard de la cheville en charge et à l’échographie chez tous les patients. Un seul patient avait bénéficié d'une exploration IRM. Le traitement, initialement médical dans tous les cas, s'est soldé par une chirurgie de résection de l'angle postéro-supérieur du calcanéum chez un patient. L’évolution a été favorable chez tous les patients.

## Introduction

Décrit par le suédois Patrick Haglund en 1928, la maladie de Haglund désigne les douleurs de l'arrière pied d'origine mécanique en rapport avec un conflit entre les différents éléments de la région rétro calcanéenne [1]. Il s'agit en fait d'un conflit pied-chaussure lié à une anomalie morphologique de la tubérosité postéro-supérieure du calcanéum avec bursite retro-calcanéenne et pré-achilléenne inflammatoire et tendinopathie achilléenne. Affection handicapante surtout chez les sportifs, il serait l'apanage du genre féminin [2] et représente une étiologie méconnue des talalgies postérieures [1]. Son diagnostic clinique est souvent source de confusion puisque le tableau clinique peut mimer d′autres causes de douleur de l′arrière-pied [3]. Nous rapportons les cas de trois patients, en présentant au travers d'une revue de littérature les caractéristiques radio-cliniques et les aspects thérapeutiques de cette pathologie.

## Patient et observation

### Cas N°1

**Figure 1:**
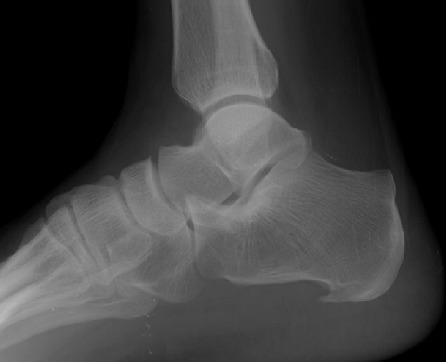
Aspect radiographique d'une proéminence de l'angle postéro-supérieur du calcanéum associée à une enthésophyte de l'aponévrose plantaire

**Figure 2:**
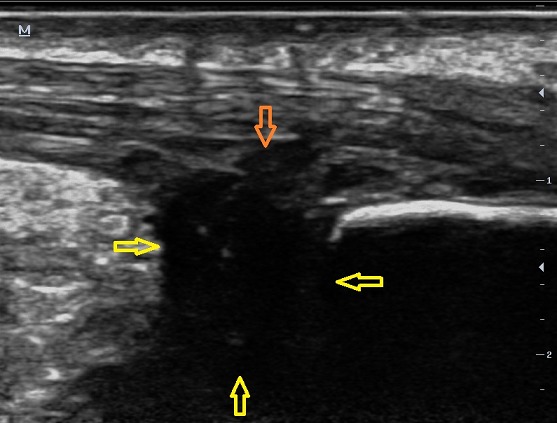
Aspect échographique d'un épanchement liquidien anéchogène pré achilléen en rapport avec une bursite pré achilléenne (flèche jaune) associée à une rupture des fibres antérieures du tendon d'Achille (flèche orange)

**Figure 3:**
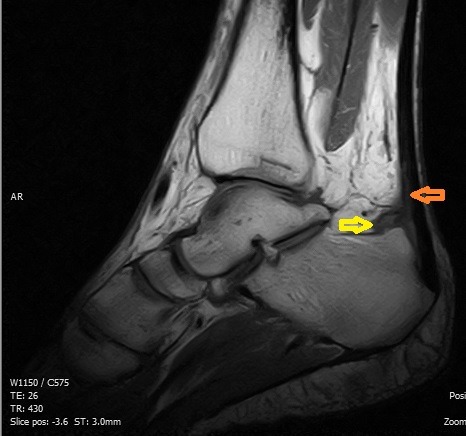
Aspect IRM, séquence sagittale T1, d'une lésion en isosignal intratendineuse (flèche orange) avec un épanchement dans la bourse préachiléenne (flèche jaune) traduisant une tendinose distale par conflit de Haglund

**Figure 4:**
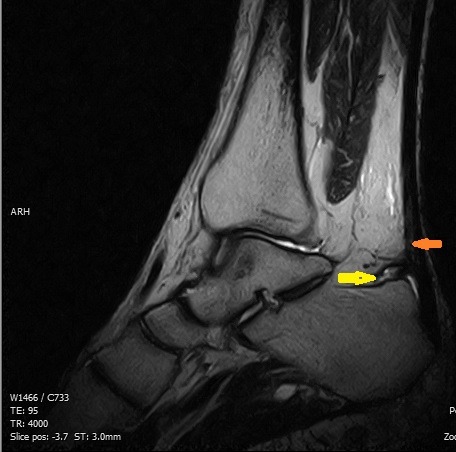
Aspect IRM, séquence sagittale T2, d'une lésion en hypersignal intratendineuse (flèche orange) avec un épanchement dans la bourse préachiléenne (flèche jaune) traduisant une tendinose distale par conflit de Haglund

Il s'agissait d'un patient de 40 ans, sans antécédent particulier, adressé pour exploration d'une tuméfaction douloureuse de l'arrière pied droit avec une talalgie postérieure extrêmement gênante, exagérée au chaussage, atténuée par le repos. Cette douleur était rebelle au traitement médical et une radiographie standard de profil en charge de la cheville a été demandée. Cet examen a objectivé une pro éminence de l'angle postéro supérieur du calcanéum associée à une enthésophyte de l'aponévrose plantaire (Figure 1). L'angle de Fowler et Philip était égal à 79^°^. L’échographie de la cheville a mis en évidence un épanchement liquidien anéchogène pré achilléen en rapport avec une bursite pré achilléenne associée à une rupture des fibres antérieures du tendon d'Achille (Figure 2). Le diagnostic d'une tendinose distale pré achilléenne (maladie de Haglund) a été évoqué. Une imagerie par résonance magnétique (IRM) a confirmé ce diagnostic en montrant un épaississement du tendon d'Achille vers la distalité mesuré à 09 mm avec des anomalies de signal intra tendineux en iso signal T1 (Figure 3) et léger hypersignal T2 (Figure 4). Il n'existait pas d'anomalie des autres ligaments de la cheville. Il s'y associait un épanchement liquidien dans la bourse pré achilléenne (Figure 3, Figure 4). L’échec du traitement médical à base d'une infiltration péritendineuse de corticoïdes (Hydrocortisone 50 mg/ 2 ml) a conduit à la réalisation d'un traitement chirurgical ayant consisté en une résection de l'angle postéro-supérieur et de l'ostéophyte inférieur du calcanéum, associée à une excision de la bourse séreuse calcanéenne. L’évolution a été favorable avec un recul de 12 mois.

### Cas N°2

**Figure 5:**
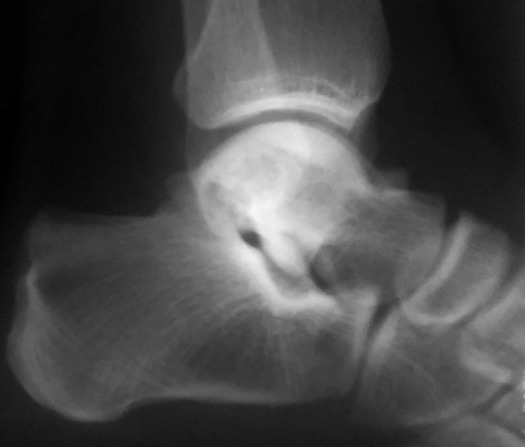
Aspect radiographique d'une proéminence de l'angle postéro-supérieur du calcanéum

**Figure 6:**
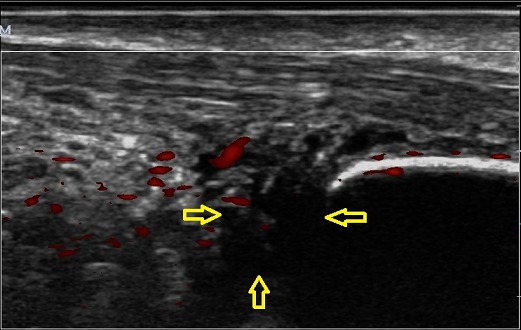
Aspect échographique d'un épanchement liquidien hétérogène à prédominance hypoéchogène pré achilléen (flèche jaune). Vascularisation des fibres antérieures du tendon d'Achille par des vaisseaux provenant de la graisse de Kager

Il s'agissait d'une patiente de 42 ans ayant présenté une douleur postérieure de la cheville gauche avec œdème évoluant depuis environ 6 mois. Une radiographie standard de la cheville réalisée de profil en charge a permis de noter une discrète pro éminence de l'angle postéro-supérieur du calcanéum avec un angle de Fowler et Philip égal à 77^°^ (Figure 5). L’échographie de la cheville montrait une collection hétérogène pré achilléenne, à prédominance hypoéchogène, avec une rupture partielle et une vascularisation des fibres antérieures du tendon d'Achille par des vaisseaux provenant de la graisse de Kager (Figure 6). La maladie de Haglund a été retenu et la patiente a bénéficié d'une infiltration péritendineuse de corticoïdes (Hydrocortisone 50 mg/ 2 ml) sous contrôle échographique. L’évolution a été favorable avec un recul de 7 mois.

### Cas N°3

**Figure 7:**
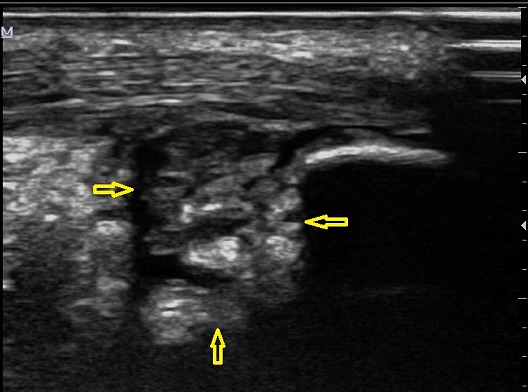
Aspect échographique d'un épanchement liquidien hétérogène pré achilléen à prédominance isoéchogène à la graisse traduisant une bursite (flèche jaune)

Il s'agissait d'un patient de 37 ans ayant présenté un œdème douloureux de la cheville droite. La radiographie standard montrait une pro éminence de l'angle postéro-supérieur du calcanéum avec un angle de Fowler et Philip mesuré à 76^°^. L’échographie de la cheville atteinte a mis en évidence une collection hétérogène pré achilléenne, à prédominance isoéchogène à la frange graisseuse de Kager traduisant une bursite préachiléenne associée à un épaississement tendineux achilléen (Figure 7). Les antalgiques et l'infiltration locale de corticoïdes (Hydrocortisone 50 mg/ 2 ml) sous contrôle échographique a permis de calmer les symptômes. Le patient est asymptomatique depuis lors avec un recul de 5 mois.

## Discussion

Les talalgies sont un symptôme de présentation commune dans les cliniques ambulatoires dont les étiologies sont nombreuses mais une étiologie mécanique reste la plus courante [4]. La maladie de Haglund en est une cause qui serait l'apanage des femmes [2]. Cependant, les travaux de Ahn et al [5] sur cette affection ont concerné 93,3% d'hommes pour 6,7% de femmes. Elle n'est donc pas si rare chez les sujets de sexe masculin puisque nos observations concernaient deux hommes pour une femme. L’âge moyen rapporté est de 33,1 ± 8,2 ans avec des extrêmes de 20 à 50 ans [5]. L’âge de nos patients, ici présentés, se retrouvent bien dans cette tranche d’âge. Nous pensons que la maladie de Haglund est souvent méconnue aussi bien par les cliniciens que par les radiologues car il faudrait y penser pour la chercher et ainsi poser le diagnostic.

Plusieurs théories étiopathogéniques (dysplasique, rhumatismale, traumatique et pied creux) ont été avancées [1, 6]. Du point de vue physiopathologique, le syndrome de Haglund est lié aux modifications morphologiques de la région rétrocalcanéenne, incriminant surtout un conflit entre la face profonde du tendon d'Achille et la tubérosité postéro-supérieure du calcanéum anormalement saillante et hypertrophiée, comme chez nos trois patients. Le tableau clinique est dominé par une tuméfaction douloureuse du talon, aggravée à la marche et aussi au chaussage et lors de la flexion dorsale, associée parfois à la présence des signes inflammatoires. Le diagnostic est fondé sur la plainte subjective et l'examen retrouve une douleur à la palpation de la proéminence calcanéenne [7]. La radiographie standard et l’échographie suffisent généralement pour poser le diagnostic [1]. La radiographie standard de profil en charge de la cheville permet d’évaluer le pied creux s'il existe et confirme la proéminence de l'angle postéro-supérieur du calcanéum, souvent sous-estimée en raison de la présence de fibrocartilage non visible à la radiographie. Des mesures angulaires radiographiques permettent d’évaluer la verticalisation du calcanéus et/ou l'importance de la proéminence (l'angle de Fowler et Philip, l'angle de Chauvaux et Liet) à l'insertion du tendon d'Achille. Cette saillie osseuse décrite par divers auteurs a été objectivée, avec une importance variable, chez nos patients. L’échographie peut mettre en évidence des signes d'irritation et de compression des tissus mous à type de bursite pré ou rétro achilléenne, avec une paroi épaisse et hyper-vascularisée au doppler couleur. Elle permet aussi l’étude du tendon d'Achille allant d'une simple tendinopathie jusqu’à la rupture (fissuration, nodule intratendineux et dégénérescence kystique et rupture) [1, 7, 8] et de faire le suivi thérapeutique des lésions tendineuses achilléennes. La bursite peut être d'aspect varié: hypoéchogène homogène ou hétérogène comme retrouvé chez deux de nos patients, voire isoéchogène et pouvant alors n’être suspectée qu'au Doppler couleur ou énergie. L'IRM, non obligatoire, plus performante que l’échographie, vient en dernière intention pour mieux analyser l’état du tendon calcanéen [1, 7, 8]. Elle avait confirmé la tendinose chez notre premier patient.

Avant de retenir le diagnostic de maladie de Haglund, il faut avant tout éliminer d'autres pathologies responsables des douleurs postérieures de la cheville et du talon. Il s'agit des tendinopathies micro-traumatiques ou métaboliques (l'hyperuricémie, les dyslipidémies), des tendinopathies inflammatoires (spondylarthrite ankylosante, polyarthrite rhumatoïde), de la fracture de fatigue du calcanéum ou d'une bursite rétro-calcanéenne isolée [1, 6, 9]. Le traitement de cette affection fait d'abord appel à des moyens médico-physiques. Le traitement médical, souvent instauré en premier, est basé sur les anti-inflammatoires non stéroïdiens et des infiltrations péri tendineuses de corticoïdes, de préférence sous contrôle échographique comme chez deux de nos patients. Les traitements locaux par mésothérapie peuvent être aussi réalisés. La rééducation (traitement physique) est axée sur le massage transversal du tendon d'Achille et l'utilisation des ultrasons et de la cryothérapie [2].

L’échec du traitement médical est fréquent, même s'il est maintenu plusieurs mois. Il est alors indiqué de recourir au traitement chirurgical [7]. Cette dernière permet d'enlever la saillie osseuse, source de conflit [1]. Deux techniques peuvent être utilisées [7]: résection de l'angle postéro-supérieur du calcanéum ou ostéotomie calcanéenne cunéiforme à base supérieure (Zadek). A la résection osseuse, il faut savoir ajouter, selon les cas, l'excision de la bourse séreuse calcanéenne ou celle des lésions tendineuses dégénératives à la face antérieure du tendon. Notre premier patient avait bénéficié d'une résection osseuse avec excision de la bourse séreuse. La calcanéoplastie endoscopique constitue un autre moyen thérapeutique dans les pays bien médicalisés [7].

Les suites opératoires n'ont aucune particularité, si non qu'il faut insister sur la talonnette post opératoire et surtout sur la suppression de tout conflit avec un contrefort agressif d'une chaussure.

## Conclusion

La maladie de Haglund est une cause souvent méconnue de talalgies postérieures. Il faut y penser, la chercher et faire son diagnostic. L'imagerie axée sur la radiographie standard et l’écho-Doppler, quelque fois sur l'IRM, permet de confirmer le conflit calcanéo-achilléen. Le traitement, initialement médical et / ou physiques, peut se solder en cas d’échec par une prise en charge endoscopique voire chirurgicale.
